# Precocious puberty or growth hormone deficiency as initial presentation in Mayer-Rokitansky-kuster-Hauser syndrome: a clinical report of 5 cases

**DOI:** 10.1186/s12887-022-03474-0

**Published:** 2022-07-14

**Authors:** Zhuanzhuan Ai, Xiaoyun Zhu, Hong Chen, Ruimin Chen

**Affiliations:** Department of Endocrinology, Genetics and Metabolism, Fuzhou Children’s Hospital of Fujian Medical University, Fuzhou, 350005 Fujian China

**Keywords:** Mayer-Rokitansky-kuster-Hauser syndrome, Children, Precocious puberty, Growth hormone deficiency, Case report

## Abstract

**Background:**

We report five patients with Mayer-Rokitansky-Küster-Hauser syndrome (MRKHS), four of whom presented with precocious puberty and one with growth hormone deficiency (GHD. Our five children add to the growing endocrine data base of MRKHS.

**Case presentation:**

We retrospectively reviewed clinical data of 5 MRKHS patients from 2017 to 2020. The clinical features, hormonal profiles, radiological imaging and genetic analyses were collated. The age range of the 5 patients at diagnosis was 6.7–9.1 years. Four presented with premature thelarche, and one presented with short stature. External genitalia were normal in all patients. Gonadotropin-releasing hormone stimulation tests for the 5 patients revealed peak luteinizing hormone and follicular stimulating hormone levels of 3.57, 6.24, 11.5, 4.44 and 4.97 IU/L and 9.41, 16.7, 13.8, 14.2 and 10.3 mIU/mL, respectively. Growth hormone stimulation for one patient with short stature was consistent with GHD with a peak level of GH was 7.30 ng/mL. Imaging disclosed advanced bone age in four patients and no skeletal abnormalities in any of the patients. Ultrasonography of the abdomen revealed bilateral polycystic kidneys in one patient. Pelvic magnetic resonance imaging confirmed no uterus in five patients. All of the patients had a normal karyotype (46, XX). In one patient, whole-exome sequencing detected a deletion of 17q12(chr17:36,046,434–36,105,050, hg19) encompassing the *HNF1B* gene.

**Conclusions:**

We report the unusual co-occurrence of precocious puberty and GHD in patients with MRKHS, highlighting that abnormal puberty and growth development may represent initial unexplained manifestations. Whether the deletion of 17q 22 begat GHD is unclear.

## Background

Mayer-Rokitansky-Küster-Hauser syndrome (MRKHS; OMIM # 277,000) is a congenital syndrome characterized by complete absence or hypoplasia of Mullerian structures, including fallopian tubes, uterus and upper two-thirds of the vagina. Notably, external genitalia are normal. MRKHS is classified into two subtypes: MRKHS type 1 is associated with isolated abnormalities of the reproductive system, whereas MRKHS type 2 also has an array of other anomalies, including renal and skeletal malformations, short stature, ontological anomalies and other defects [1]. MRKHS was first described by Mayer in 1829 with an estimate female incidence of 1 in 5,000 live births [2]. The molecular basis of MRKHS remains elusive, although a few candidate genes are considered likely.

Generally, patients affected with MRKHS have a normal (46, XX) karyotype, as well as normal ovarian function and age-appropriate secondary sex characteristics (breast growth, body hair, body proportions) [3]. Typically, MRKHS girls are clinically healthy and are not identified until late puberty due to absent of menarche or dyspareunia [1]. To date, endocrine function in MRKHS has been inadequately investigated, but allegedly endocrine function is intact [4]. Herein, the presenting endocrine disorders of five Chinese children with MRKHS are delineated.

## Case presentation

Detailed data from five MRKHS patients from July 2017 to December 2020 in the Fuzhou Children’s Hospital of Fujian Medical University was collated. This study was reviewed and approved by the Ethics Committee of Fuzhou Children’s Hospital of Fujian and was conducted in agreement with the Declaration of Helsinki Principles. Each of the patients and parents gave informed consent.

### Clinical evaluations

During the initial examination, the five patients were assessed by medical history and clinical manifestations. Growth and development evaluation height, weight and body mass index (BMI), Tanner staging for girls were determined by clinical specialists to evaluate the stage of puberty development. Bone age determination was done by the Tanner-Whitehouse 3 (TW3) method. Relevant laboratory examination included follicle-stimulating hormone, luteinizing hormone, estradiol and growth related hormones.

After overnight fasting, the stimulation test was commenced at 9:00. Sampling was done at 0, 30, 60, 90, and 120 min. Arginine (10 mg/kg) and levodopa (10 mg/kg) were administered for the growth hormone (GH) secretion test. GH deficiency (GHD) was defined by a peak GH value less than10 ng/ml.

Provocation testing with GnRH Gonadorelin was administered intravenously according to a dosage level of 2.5 µg/kg (maximum dose, 100 μg). Serum LH and FSH levels were assayed at 0, 30, 60, 90 and 120 min after the injection using a chemiluminescence-based method. A peak stimulated LH level of > 3.3 ~ 5.0 U/L is considered diagnostic of central precocious puberty (CPP), and a ratio of LH/FSH > 0.6 is also consistent with this diagnosis.

### Case 1

**Fig. 1:**
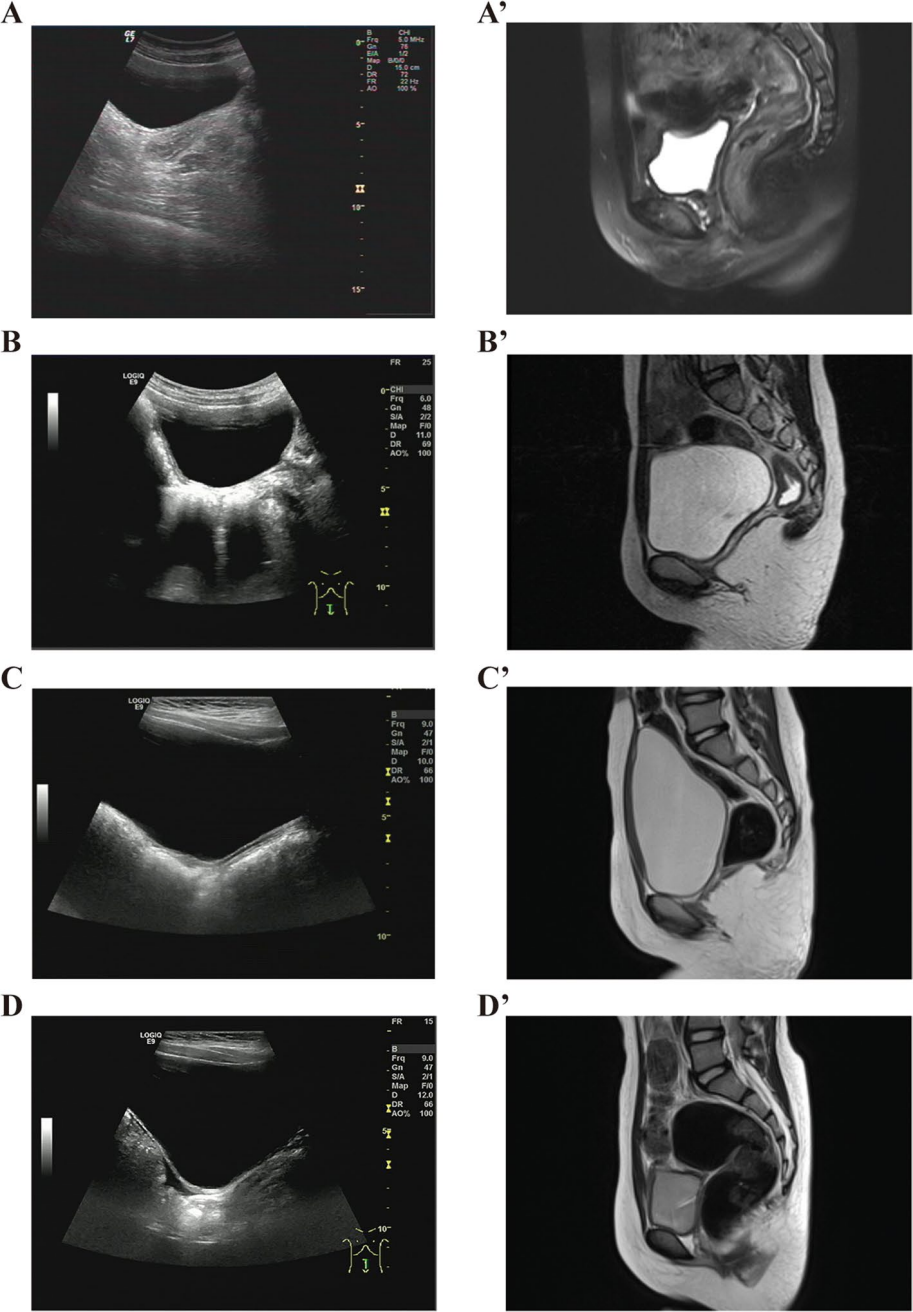
The pelvic ultrasound and MRI of case 1-case 4. **A** The pelvic ultrasound of case 1. **A’** The pelvic MRI of case 1. **B** The pelvic ultrasound of case 2. **B’** The pelvic MRI of case 2. **C** The pelvic ultrasound of case 3. **C’** The pelvic MRI of case 3. **D** The pelvic ultrasound of case 4. **D’** The pelvic MRI of case 4

A 6-year-11-month-old girl was admitted for premature thelarche. She was the second child born at full term by cesarean section with a birth weight (BW) of 2.9 kg(-1.3SD) and a birth length (BL) of 50 cm(+ 0.2SD). There was no antenatal history of viral or bacterial infection nor any chronic illness during gestation. There were no significant developmental problems, and no history of consanguinity. On physical examination, breast development was Tanner stage 2, pubic hair and axillary hair development Tanner stage 1. Her height was 128.1 cm [+ 1.24 standard deviation score (SDS)] cm and weight 25.5 (+ 0.13SDS) kg. Her BMI was 15.5 kg/m^2^. External genitalia were normal, and she did not undergo internal pelvic examination due to virginity, the rest of the exam was normal. Routine hematological and biochemical analyses were normal. Endocrine evaluation revealed a basal luteinizing hormone (LH) < 0.1I U/L and basal follicle stimulating hormone (FSH) of 2 IU/L, Gonadotropin-releasing hormone (GnRH) stimulation test revealed a peak LH level of 3.57 IUI/L and peak FSH 9.95 IU/L with an estradiol (E2) 5 pg/mL (reference range, 0–5 pg/mL). Additional hormone assays included a testosterone (T) of 2.5 ng/dl (reference range, 0–2.5 ng/dl) and dehydroepiandrosteron (DHEA) level of 80.9 ug/dl (reference range, 35–430 ug/dl). The bone age (BA) was 9.1 years using the Tanner-Whitehouse 3 (TW3) method, and predicted height was 154 ± 5 cm. Her karyotype was 46, XX and the results of whole-exome sequencing (WES) were negative. A radiographic examination of the spine was normal. The suspected size of both ovaries was about 1.7 cm × 1.2 cm × 1.0 cm (1.02 mL). An absent uterus which was confirmed by MRI (Fig. 1 A, A’). Brain MRI revealed a Rathke cyst. Based on all these findings, she was assigned the clinical diagnosis of MRKHS with incomplete precocious puberty.

### Case 2

A 7-year-9-month-old girl was evaluated because of recent breast enlargement. She was the first child born at full term by spontaneous delivery with a BW of 2.9 kg(-1.3SD)and a BL of 50 cm(+ 0.2SD). There was no antenatal history of any infection or chronic illness during gestation. She had no significant developmental problems and her family history was negative with no consanguinity. On physical examination, she had Tanner stage 2 breast development and Tanner stage 1 pubic hair. Her height was 128.5 cm (+ 0.26 SDS) and weight 24 kg (-0.38 SDS). Her BMI was 14.5 kg/m^2^. External genitalia were normal and she did not undergo internal pelvic examination due to virginity. The remainder of her examination was normal. Laboratory results were as follows: basal LH 0.27 IU/L, basal FSH 3.9 IU/L, GnRH stimulation test had a peak LH level of 6.24 IUI/L, peak FSH 16.7 IU/L, E2 < 5 pg/mL, T 4.48 ng/dl and DHEA level 33.2 ug/dl. Routine hematological and biochemical analyses were normal. Her BA was 8.1 years using the TW3 method and predicted height was 160 ± 5 cm. Her karyotype was 46, XX and the results of WES and copy number variation (CNV) sequencing were likewise negative. Spine X-ray was normal. Abdominal-pelvic ultrasound and MRI showed ovarian of pubertal volumes (right ovary: 2.2 × 1.3 × 1.2 cm; left ovary: 2.2 × 1.4 × 1.3 cm) and absent uterus (Fig. 1B, B’). Other visceral organs were normal. She had a Rathke cyst by brain MRI. The clinical diagnosis was consistent with MRKHS with incomplete precocious puberty.

### Case 3

An 8-year-4-month-old girl was evaluated for breast enlargement that began six months earlier. She was the first child born at 32 weeks by cesarean section with a BW of 1.3 kg(-1.5SD)and a BL of 42 cm(0.0SD). Mother was healthy during pregnancy. She began to raise her head at 4 months, sat at 7 months, spoke at 12 months and walked at 14 months. She had no cognitive impairment and no family history of consanguinity. Physical examination revealed Tanner 2 breast development, Tanner stage 1 for pubic hair. Her height was 128.7 cm (-0.31SDS) and weight 26.5 kg (+ 0.42 SDS). Her BMI was 16.0 kg/m^2^. External genitalia were normal and she did not undergo pelvic examination due to virginity.

Laboratory findings were as follows: basal LH 0.19 IU/L, basal FSH 2.77 IU/L, GnRH stimulation test had a peak LH level of 11.5 IUI/L, peak FSH 13.8 IU/L, E2: 5 pg/mL and DHEA 122 ug/dl. Routine hematological and biochemical analyses were normal. The BA was 9.7 years using the TW3 method and predicted height was 152 ± 5 cm. She had a normal karyotype (46, XX) and negative results of WES and CNV sequencing. The spine X-ray was normal. Abdominal-pelvic ultrasound and MRI revealed ovary of pubertal volume (right ovary: 2.0 × 1.0 × 0.9 cm; left ovary: 1.9 × 1.1 × 1.0 cm) and dysplasia of the uterus (Fig. 1 C, C’). Brain MRI revealed a pineal cyst. The clinical diagnosis was MRKHS with CPP.

### Case 4

A 7-year-9-month old girl was referred for premature development of breast and pubic hair. She was the first-born child, born at full term by cesarean section with a BW of 3.36 kg(+ 0.2SD)and a BL of 50 cm(+ 0.2SD). Her family history was negative and there was no consanguinity. She was Tanner stage 2 for breast and pubic hair development. Her height was 136.3 cm (+ 1.93SDS) and weight 31 kg (+ 0.55SDS). Her BMI was 16.7 kg/m^2^. External genitalia were normal and she did not undergo a pelvic exam. Laboratory results were as follows: basal LH 0.11 IU/L, basal FSH 0.69 IU/L, GnRH stimulation test had a peak LH level of 4.44 IUI/L, peak FSH 14.2 IU/L, E2 < 5 pg/mL and DHEA of 62.1 ug/dl. Routine hematological and biochemical analyses were normal. The BA was 10.7 years using the TW3 method and predicted height was 152.1 ± 5 cm. The karyotype was normal (46 XX) and the result of CNV sequencing was negative. Spine X-ray was normal. Abdominal-pelvic ultrasound and MRI excluded adrenal masses and revealed ovaries of pubertal volume (right ovary: 2.9 × 1.6 × 1.6 cm; left ovary: 3 × 1.7 × 1.5 cm) and absent uterus (Fig. 1 D, D’). The remainder of the exam was normal. Brain MRI was negative. The clinical diagnosis was consistent with MRKHS with incomplete precocious puberty.

### Case 5

**Fig. 2:**
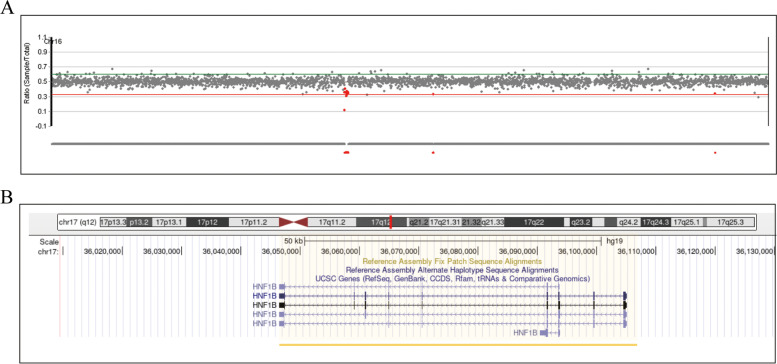
Whole-exome sequencing (WES) analysis of the case 5. **A** The vertical axis is the signal ratio between the sample and the standard sample. **B** Schematic representation of chromosome region 17q12 using UCSC Genome Browser assembly February 2009 hg19. The missing areas in our patient were chr17:36,946,434–36,105,050

**Fig. 3:**
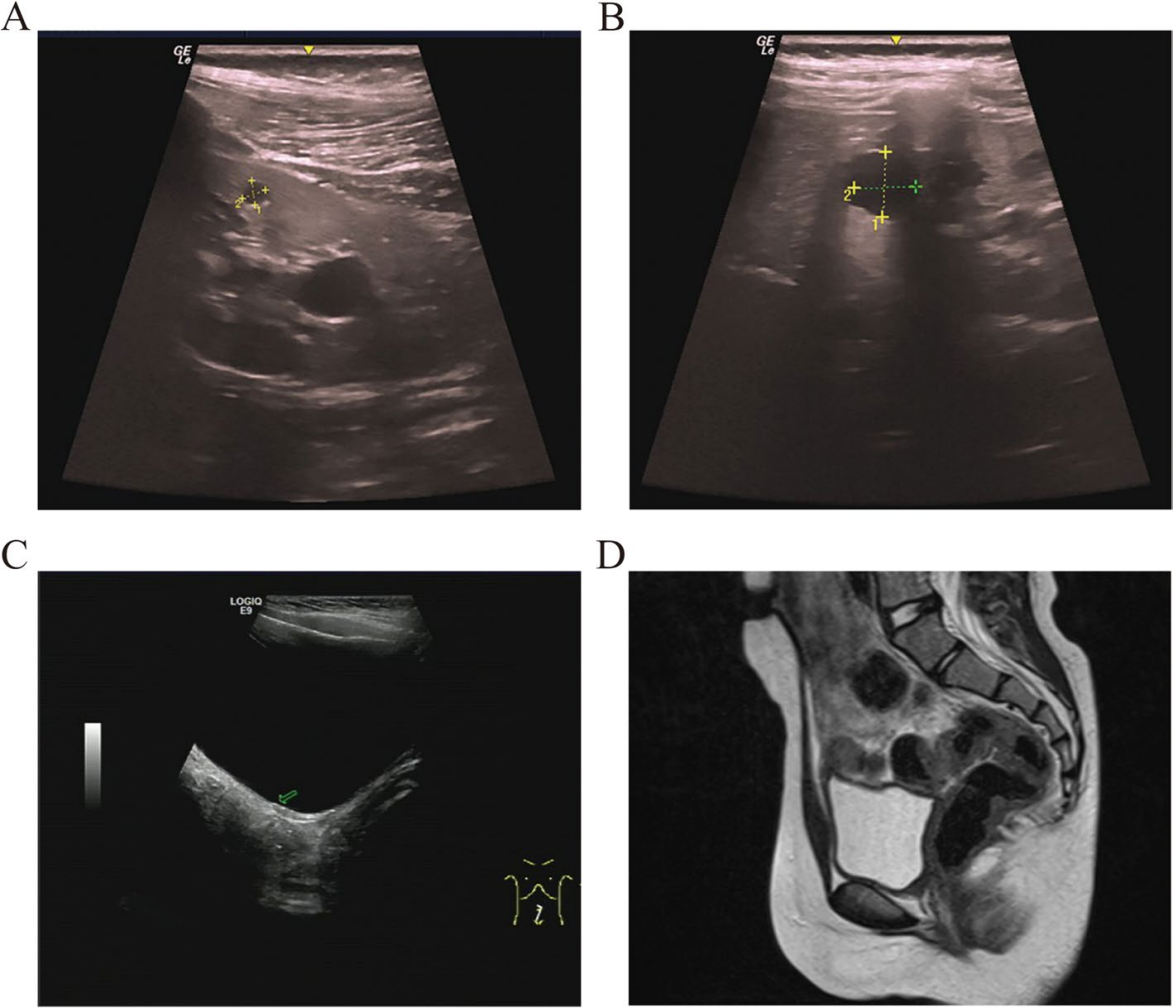
The pelvic ultrasound and MRI of case 5. **A**, **B** Ultrasonography of the kidneys. Numerous cysts are scattered in the kidney. **C** The pelvic ultrasound of case 5. **D** The pelvic MRI of case 5

A 9-year-2-month-old girl was referred to our hospital for chronic growth retardation and onset of thelarche(less than one year). She was the first child born at full term by cesarean section. Her BW was 3.2 kg(+ 0.5SD), BL 49.0 cm(+ 0.0SD). She had no psychomotor or cognitive developmental issues. Maternal height was 162 cm father was 168 cm. Her family history was negative and there was no consanguinity. She had breast stage 2, pubic hair stage 1. Her height and weight were120.3 cm (− 2.5 SDS) and 21.5 kg (− 0.1 SDS), respectively. Her BMI was 14.9 kg/m^2^. External genitalia were normal. She did not undergo a pelvic examination due to virginity. Laboratory parameters were as follows: basal LH < 0.1 IU/L, basal FSH 1.35 IU/L, GnRH stimulation test had a peak LH level of 4.97 IUI/Land peak FSH 13.8 IU/L. Her GH in response to a growth hormone (GH) stimulation test(levodopa and clonidine) was 7.63 ng/mL (normal: ≥ 10 ng/mL), E2 < 5 pg/ml. In addition, insulin-like growth factor 1, insulin-like growth factor binding protein 3 and insulin levels were all in the normal range. Routine hematological and biochemical analyses were normal. Her BA was 8.75 years according to the TW3 method. Her karyotype was normal, and WES analysis confirmed a deletion of 17q12 (chr17:36,046,434–36,105,050, hg19) (Fig. 2 A), encompassing the *HNF1B* gene (Fig. 2 B). Abdominal-pelvic ultrasound disclosed bilateral polycystic kidneys (Fig. 3 A, B), ovaries of pubertal volume (right ovary: 3.3 × 1.2 × 1.0 cm; left ovary: 3.0 × 1.4 × 1.1 cm) and absent uterus (Fig. 3 C). Pelvic MRI confirmed the absence of uterus (Fig. 3 D). No remarkable findings were observed on the spine X-ray and pituitary MRI. The clinical diagnosis was MRKHS with GHD and advanced puberty. After treatment with growth hormone therapy (0.12 IU/kg) for one year, her height gained 10.4 cm (-1.75 SDS).

## Discussion and conclusion

MRKHS is an uncommon developmental disorder and is often diagnosed due to primary amenorrhea in late puberty, whereas some patients are coincidentally identified in early infancy due to associated anomalies or unrelated ailments [5]. Once the diagnosis of MRKHS is suspected, confirmation is based on further investigation including imaging (Ultrasound/MRI), karyotyping/chromosome analysis and serum hormone status. MRI is considered to be the preferred procedure, and is a more suitable option in pediatric patients [6]. All five of our patients fulfilled the clinical diagnostic criteria for MRKHS. It is noteworthy that both advanced puberty and GHD occurred in one patient, which is quite distinct from the typical presentation of MRKHS. The clinical characteristics and management of these patients are summarized in the Table 1. We painstakingly reviewed the literature and found that this tandem hormone abnormality (PP, GHD) is atypical. Only two patients with PP in MRKHS have been previously reported [7, 8], and patient with GHD has never been reported.

**Table 1:** Clinical and biochemistry characteristics of the 5 patients

	Case 1	Case 2	Case 3	Case 4	Case 5	Normal ranges
Age at onset(year)	6 5/12	7 9/12	7 10/12	7 9/12	2/12	
Age at Diagnosis(year)	6 11/12	7 9/12	8 4/12	7 9/12	9 2/12	
Gender	F	F	F	F	F	
Family history	Negative	Negative	Negative	Negative	Negative	
BW (kg)	2.9	2.9	1.3	3.36	3.2	
BL (cm)	50	50	42	50	49	
Height (cm)	128.1	128.5	128.7	136.3	120.3	
z-score	1.24	0.26	-0.31	1.93	-2.5	
Predicted height(cm)	154 ± 5	160 ± 5	152 ± 5	152.1 ± 5	__	
Weight (kg)	25.5	24	26.5	31	21.5	
BMI (kg/m^2^)	15.56	14.65	16.17	16.76	14.93	
BA (years)	9.1	8.1	9.7	10.7	8.75	
Tanner staging	B2P1	B2P1	B2P1	B2P2	B2P1	
Laboratory test						
LH peak (IU/L)	3.57	6.2	11.5	4.44	4.97	
FSH peak (IU/L)	9.95	16.7	13.8	14.2	10.3	
GH peak(ng/ml)	—	—	—	—	7.3	
T (ng/ld.)	2.5	4.48	7.63	< 2.5	—	0–2.5
E2 (pg /ml)	5	< 5	5	< 5	< 5	0–5
ACTH (pg/ml)	< 5	9.51	11.4	13.8	9.74	0–46
Cortisol (nmol/L)	290	159	174	684	204	138–690
PROG (ng/ml)	0.29	< 0.2	< 0.2	1.35	—	0–1.4
17-OHP (nmol/L)	0.9	1.4	0.6	3	—	0–11.5
DHEA (ug/dl)	80.9	33.2	122	62.1	—	35–430
SHBG(nmol/L)	—	—	—	—	87.8	
AND (ng/ml)	< 0.3	0.91	—	0.79	—	0.3–3.5
PRL (ng/ml)	20.1	10.4	10.9	26.1	4.27	
hCG (mIu/ml)	< 1	< 1	< 1	< 1	< 1	0–2.7
Karyotype	46, XX	46, XX	46, XX	46, XX	46, XX	
mutation	Negative	Negative	Negative	Negative	17q12 deletion	
Image						
Pelvic ultrasound						
Right over volume (m^3^)	1.02	1.72	0.9	3.71	1.98	< 2.0
Left over volume (m^3^)	1.02	2	1.05	3.82	2.31	< 2.0
Uterus	Absent	Absent		Absent	Absent	
Pelvic MRI(uterus)	Absent	Absent	Absent	Absent	Absent	
renal ultrasound	—	—	—	—	renal cystic	
Brain MRI	Rathke cyst	Rathke cyst	Negative	Pineal cyst	Negative	
Therapy	—	—	—	—	rhGH	

*ACTH* Adrenocorticotropic hormone, *AND* Androstenedione, *BA* Bone age, *BMI* Body mass index, *BW* Birth weight, *BL* Birth length, *DHEA* Dehydroepiandrosteron, *E2* Estradiol, *F* Female, *FSH* Follicular stimulating hormone, *GH* Growth hormone, *hCG* Human chorionic gonadotropin, *SDS* Standard deviation score, *LH* Luteinizing hormone, *PROG* Progesterone, *PRL* Prolactin, *rhGH* Recombinant human growth hormone, *SHBG* Sex hormone-binding globulin, *T* Testosterone, *17α-OHP* 17-α-hydoxy progesterone, *MRI* Magnetic resonance imaging

There are two major types of MRKH syndrome. Type I is characterised by congenital aplasia of the uterus and upper two-thirds of the vagina. Type II also incorporates extragenital/extra-Müllerian malformations, including vertebral, cardiac, urological (upper tract) and ontological anomalies [9]. The MRKHS phenotype is highly variable. Poorly developed, or agenesis of vagina or uterus can be found. The ovaries usually arise from a separate embryologic source (urogenital ridge), and are generally normal, although abnormal ovarian function and location have been reported [10, 11]. A significant proportion of MRKHS patients have associated extragenital malformations. Of these, renal malformations are the most prevalent associated anomalies (~ 33%) [2], incorporating a wide spectrum that includes renal agenesis, horseshoe kidney, pelvic kidney and renal cysts. In our study, bilateral polycystic kidneys were found in one patient. A prevailing theory for renal malformations posits the close physical and temporal proximity development process between Mullerian and Wolffian ducts [12]. A host of other MRKHS malformations are as follows: skeletal anomalies (e.g., vertebral arch disturbances, scoliosis, hypoplasia of the wrist, bilateral tibial longitudinal deficiency, hypoplastic sternum), hearing impairment, cardiac malformations, and umbilical/diaphragmatic/abdominal wall hernia. Co-existing anomalies account for the clinical heterogeneity and complexity of MRKHS. The VCUAM (vagina, cervix, uterus, adnexae-associated malformation) system is the consensus classification to categorize the associated malformations in MRKHS [13]. In our series, only one patient could be classified as type 2.

The congenital nature and multiple reports of familial aggregates implicate a genetic etiology [14]. A deletion at chromosome 17q12, spanning more than 100 kb of genomic DNA and containing *HNF1B,* was detected in one patient. The 17q12 region genes is a relatively common CNV in both MRKHS types 1 and 2 [15]. Deletions of 17q12 range from 1.2–1.8 Mb in size, and no association between disparate deletion sizes and phenotypic have been discerned [16]. *HNF1B* resides within this region and could plausibly be a promising genetic candidate for MRKHS [17]. This gene is highly expressed in Wolffian and Müllerian ducts and plays a vital role in the development and differentiation of liver, kidney, pancreas and genital tract [15]. *HNF1B* haploinsufficiency contributes to impairment of uterine development by down-regulating key genetic factors involved in urogenital development, such as *LHX1, PAX2,* and *WNT9B* [18]. Heterozygous mutations or whole gene deletions of *HNF1B* gene are associated with variable renal abnormalities, such as renal cysts, and hypo-dysplasia [19]. However, these genetic abnormalities are not described in isolated uterine defects [20]. No mutations in *HNF1B* have been detected with MRKHS [21]. Although 17q12 CNV has been reported in MRKHS, other mutated genes within this region may be causative.

Due to the complex clinical characteristics and etiology of MRKHS, childhood presentation can vary. Four patients in our study presented with premature thelarche and advanced BA, and premature pubarche in one of them. Notably, early onset of puberty is the cardinal feature in our report, distinct from the typically initial adult manifestation of MRKHS. A GnRH test was performed which confirmed CPP in one patient (case 3) and incomplete precocious puberty confirmed in three patients. PP accounts for the bone maturation and reduced final adult height. To our knowledge, only two MRKHS with PP have been previously reported. The first patient was reported in 2001 by Raybaud et al. [7], who described a 8 year-8 month- old girl with obesity, CPP and type 1 MRKHS. The other patient was a 7-year-old girl with urinary incontinence, CPP and type 2 MRKHS ((vaginal agenesis, absence of uterus and single pelvic kidney) [13]. The most common etiology of PP is the premature activation of the hypothalamic-pituitary–gonadal (HPG) axis. Pineal cyst and Rathke cyst were detected by MRI in three of our patients. Recently, the prevalence of pineal cyst (0.8%) and Rathke cyst (2%) have been reported in 251 girls with CPP [22]. One conceivable explanation is that a cyst somewhat abrogates the normal prepubertal gonadotropin inhibition [23, 24]. The pathogenetic mechanism for PP in MRKHS, however, remains elusive. A normal steroid hormone interaction between the ovaries and uterus is essential for normal regulatory mechanisms, especially during the menstrual cycle [25]. Strissel et al. [26] proposed that the normal ovarian-uterine steroid communication is disrupted in MRKHS. Accordingly, we conjecture that the missing crosstalk between ovary and uterus may disturb the hypothalamic feedback system, thereby promoting premature gonadotropins secretion. Indeed, a significantly higher levels of LH and FSH are observed in MRKHS compared to controls [27], and an increased incidence of aberrant hormone levels in patients with MRKHS, including hyperprolactinemia and hyperandrogenemia, further support our hypothesis [27, 28]. Taken together, based on the patients reported in this study along with those previously published, we opine that early puberty development in MRKHS is not uncommon.

Short stature, a common malformation in the type II MRKHS, was found in one of our five patients. GH stimulation tests revealed GHD. Although short stature is known to be associated with type II MRKHS, the underlying etiology is not fully understood. Both skeletal anomalies and partial duplication of pseudoautosomal Xpter region 1, containing the SHOX gene for short height, could be responsible [2, 29]. Our patient presented with both GHD and early puberty. The pathogenesis of GHD is unclear. Studies of additional MRKHS patients may determine the factors that disturb ancillary hypothalamic-pituitary pathways beyond the HPG axes. Moreover, it is noteworthy that a postnatal growth reduction has been reported in patients with *HNF1B* deletion and *HNF1B*-knockout mice, however, whether this is due to growth hormone deficiency is uncertain [30, 31].

Information related to gonadotrophin releasing hormone agonist (GnRHa) or GH therapy in patients with MRKHS are lacking. Until now, only one patient with PP has been reported in a child with MRKHS who was treated with GnRHa [7]. As for GH therapy, twelve months of GH treatment in a short MRKHS patient induced a significant increase in height without complications [32]. In this study, we did not prescribe GnRHa to the patient with PP because she had a normal predict height notwithstanding her advanced BA. Our patient is currently receiving rhGH therapy and given the positive growing response without adverse effects, we are optimistic about her final height.

In summary, four girls with MRKHS had precocious puberty, and one had GHD. The distinctive finding is the commonality of abnormal puberty onset and growth progression in MRKHS. Although it remains unclear whether the deletion of 17q22 is involved in GHD, we opine that the endocrinological disorders in our five patients may not be a stochastic phenomenon and may be more common than realized. Further study in a larger number of MRKHS children would clarify the pathophysiology of abnormal puberty onset and growth progression.

## Data Availability

The datasets used and/or analysed during the current study are available from the corresponding author on reasonable request.

## Abbreviations

BA: Bone age
BL: Birth length
BMI: Body mass index
BW: Birth weight
CNV: Copy number variation
CPP: Central precocious puberty
DHEA: Dehydroepiandrosteron
E2: Estradiol
FSH: Follicular stimulating hormone
GH: Growth hormone
GHD: Growth hormone deficiency
GnRH: Gonadotropin-releasing hormone
HPG: Hypothalamic-pituitary–gonadal
LH: Luteinizing hormone
MRKHS: Mayer-Rokitansky-Küster-Hauser syndrome
SDS: Standard deviation score
TW3: Tanner-Whitehouse 3
